# Disadvantage of Viable Portal Vein Tumor Thrombosis in Liver Transplantation for Advanced Hepatocellular Carcinoma

**DOI:** 10.3390/cancers17020188

**Published:** 2025-01-08

**Authors:** Kun-Ming Chan, Yin Lai, Hao-Chien Hung, Jin-Chiao Lee, Chih-Hsien Cheng, Yu-Chao Wang, Tsung-Han Wu, Chen-Fang Lee, Ting-Jung Wu, Hong-Shiue Chou, Wei-Chen Lee

**Affiliations:** Department of General Surgery, Chang Gung Transplantation Institute, Chang Gung Memorial Hospital at Linkou, Chang Gung University College of Medicine, Taoyuan 33302, Taiwan; mr1709@cgmh.org.tw (Y.L.); mp0616@cgmh.org.tw (H.-C.H.); b9302012@cgmh.org.tw (J.-C.L.); chengcchj@cgmh.org.tw (C.-H.C.); b9002072@cgmh.org.tw (Y.-C.W.); domani@cgmh.org.tw (T.-H.W.); lee5310@cgmh.org.tw (C.-F.L.); wutj5056@cgmh.org.tw (T.-J.W.); chouhs@cgmh.org.tw (H.-S.C.); weichen@cgmh.org.tw (W.-C.L.)

**Keywords:** hepatocellular carcinoma, portal vein thrombosis, tumor thrombosis, liver transplantation, outcomes

## Abstract

Background: Liver transplantation (LT) is a promising treatment option for patients with hepatocellular carcinoma (HCC) comorbid with cirrhosis. However, HCC with portal vein tumor thrombosis (PVTT) remains an absolute contraindication for LT. This study aimed to analyze the outcomes of LT in patients with HCC plus portal vein thrombosis and further evaluate the impact of PVTT on the long-term outcomes of patients. Methods: Among the 501 patients who underwent LT for HCC between January 2000 and March 2023, 29 (5.8%) patients with HCC who had portal vein thrombosis were further analyzed. Of these 29 patients with portal vein thrombosis, 12 (41.4%) were preoperatively diagnosed with PVTT and underwent LT after receiving downstaging therapy. The remaining 17 (58.6%) patients were PVTT-free prior to LT. Results: Overall, the recurrence-free survival rates at 1, 3, and 5 years were 96.3%, 74.2%, and 74.2%, respectively, while the 1-, 3-, and 5-year overall survival rates were 82.4%, 74.2%, and 70.1%, respectively. However, patients with viable PVTT had significantly worse outcomes than those without viable PVTT (*p* = 0.030). The 5-year recurrence-free and overall survival rates for patients with viable PVTT were 57.5% and 57.0%, respectively. Conclusions: LT may still be a promising option for patients with HCC and PVTT after appropriate downstaging. However, caution should be adopted, as remnant viable PVTT might lead to unsatisfactory outcomes after transplantation.

## Simple Summary

Liver transplantation (LT) is an optimal treatment option for hepatocellular carcinoma (HCC) associated with cirrhosis. Additionally, the transplantation community is eager to expand the criteria for transplantation to accommodate more patients with HCC. Despite numerous efforts to broaden LT eligibility, HCC with portal vein tumor thrombosis (PVTT) remains a contraindication due to the associated risk of poor prognosis after transplantation. However, there have been arguments addressing the dilemma of LT in cases of HCC with PVTT. This study aimed to analyze the outcomes of LT in patients with HCC and portal vein thrombosis, specifically evaluating the impact of PVTT on long-term outcomes. The results indicate that the presence of residual viable tumor within the portal vein thrombus may lead to poor outcomes following LT. Therefore, patients with HCC and PVTT could still be considered for LT after appropriate downstaging treatment. However, caution should be exercised regarding the potential for residual viable PVTT, which may result in unsatisfactory outcomes after LT.

## 1. Introduction

Hepatocellular carcinoma (HCC) is one of the leading causes of cancer-related deaths globally, and often occurs in patients with underlying cirrhosis. Liver transplantation (LT) is considered an optimal treatment option for HCC as it completely removes the tumor and eliminates the underlying cirrhotic liver with a microenvironment tendency for tumorigenesis. However, the current criteria for LT in HCC are highly restrictive and exclude many patients with advanced-stage HCC [1,2]. Therefore, many expanded criteria have been introduced by numerous transplantation centers worldwide to accommodate more patients with HCC [3,4]. Despite these efforts to broaden the criteria for LT, HCC with macroscopic vascular invasion remains a contraindication for LT due to the risk of poor prognosis after transplantation [5,6]. Recently, arguments have been raised to address the dilemma of LT contraindication in HCC comorbid with portal vein tumor thrombosis (PVTT) [7].

However, thrombosis within the portal venous system may also occur because of underlying cirrhosis, complicating the decision-making process for LT in patients with HCC and thrombosis within the portal vein. Specifically, it may be challenging to differentiate between benign thrombosis and tumor thrombus when thrombosis develops in the portal vein of patients with HCC. Consequently, PVTT is often only incidentally discovered in explanted livers on histological examination, following strict transplantation criteria. Additionally, patients with advanced HCC comorbid with PVTT may be eligible for transplantation following successful downstaging to meet LT criteria [8,9,10,11]. Therefore, this study aimed to analyze the outcomes of LT in patients with HCC and portal vein thrombosis (PVT) and specifically evaluate the impact of PVTT on long-term survival.

## 2. Materials and Methods

### 2.1. Patients

A total of 1370 patients who underwent LT between January 2000 and December 2023 at the Transplantation Institute of Chang Gung Memorial Hospital in Linkou, Taiwan, were retrospectively reviewed and approved by the Institutional Review Board (IRB No.: 202301238B0). Of these, 501 patients underwent LT for HCC, including 29 with HCC comorbid with PVT at the time of transplantation. Clinical demographic and characteristics of the 501 patients composing the study population are summarized in Table 1. Subsequently, those 29 patients with PVT were enrolled for further analysis. This study adhered to the ethical guidelines established by the Declaration of Helsinki regarding confidentiality, and the requirement for informed consent was waived owing to the retrospective nature of the study.

### 2.2. Management of HCC

HCC diagnosis followed the guidelines proposed by the European Association for the Study of the Liver and the American Association for the Study of Liver Diseases [12,13]. The treatment of HCC was mainly multidisciplinary and determined by the consensus of liver cancer committee of the institute, following the Barcelona Clinic Liver Cancer treatment algorithm [6,14]. Curative treatments, such as locoregional ablation and surgical resection, were prioritized for early-stage HCC. LT was recommended for patients with liver cirrhosis and unresectable HCC who met the University California of San Francisco (UCSF) criteria based on imaging examinations [2]. Specifically, HCCs associated with major vascular invasion were excluded from LT. Distinguishing PVTT may require the need to gather information, including laboratory tests and imaging characteristics. Theoretically, PVTT would be highly suspected if the imaging features reveal an expanding thrombus with vessel wall disruption, arterial enhancement, and HCC adjacent to venous thrombosis and/or serum alpha-fetoprotein (AFP) > 1000 ng/mL.

Patients with HCC beyond the UCSF criteria typically underwent downstaging treatment first. Those who were successfully downstaged to meet the transplantation criteria—specifically regarding tumor status, with no evidence of vascular invasion, and who remained stable for at least three months—became eligible for LT. The institute adopted a shared decision-making approach to assess the willingness of patients to undergo LT [15]. Therefore, patients with HCC were placed on the waitlist only if they accepted LT as a treatment option. Moreover, patients may wait for deceased donor liver transplantation (DDLT) on the waitlist or opt for living donor liver transplantation (LDLT) when a suitable living donor is available. Additionally, some patients may initially wait for DDLT but switch to LDLT as soon as a living donor becomes available.

### 2.3. LT and Follow-Up

All liver transplants, including donor and recipient surgeries, were performed using a previously described conventional approach [16,17]. HCC tumor characteristics were determined by pathological examination of the explanted liver. Immunosuppression therapy consisted of a combination of steroids, tacrolimus, and mycophenolate mofetil. None of the recipients received induction immunosuppression therapy, and steroids were tapered off and discontinued within three months.

Patients were monitored using serum alpha-fetoprotein levels and liver ultrasonography at intervals not exceeding 3 months. Computed tomography and/or magnetic resonance imaging was performed at 6-month intervals or whenever there was clinical suspicion of HCC recurrence. However, tissue biopsy is indicated only in cases with equivocal imaging findings.

### 2.4. Outcome and Statistical Analysis

The primary outcomes were HCC recurrence-free survival (RFS) and overall survival (OS). RFS was calculated from the date of LT to HCC recurrence, and OS was calculated from the date of LT to death or the end of the study period. Survival curves were constructed using the Kaplan–Meier method and compared using the log-rank test. Categorical variables were compared using χ^2^ or Fisher’s exact test, as appropriate, while continuous variables were compared using Student’s *t*-test. Multivariate analysis was not performed because of the small sample size. Statistical analyses were performed using the SPSS software package (version 25.0; SPSS, Inc., Chicago, IL, USA) for Windows, and *p* < 0.05 was considered statistically significant.

## 3. Results

### 3.1. Clinical Features of Patients

A total of twenty-nine patients, comprising twenty-four males and five females, were included in this study according to the defined inclusion criteria. The median age of the patients was 55.1 years (range: 42.1–68.6 years). The majority of the patients (*n* = 18, 62.1%) had Hepatitis B virus-related cirrhosis, while seven (24.1%) patients were infected with Hepatitis C virus. Additionally, three patients (10.3%) were associated with an alcoholic disposition. Among these patients, 12 (41.4%) were preoperatively diagnosed with PVTT based on imaging examinations and subsequently underwent downstaging therapy. The remaining 17 (58.6%) patients were free of tumor thrombosis in the portal vein prior to LT. Patients with PVTT were treated with stereotactic body radiation or proton therapy targeting the tumor thrombus to downstage HCC to meet the transplantation criteria. Additionally, two of these patients received systemic therapy with tyrosine kinase inhibitors and immune checkpoint inhibitors (ICIs) later in their treatment. Locoregional therapies, including transarterial chemoembolization and radiofrequency ablation, were used to treat intrahepatic HCC prior to LT. One patient had previously undergone liver resection for primary HCC but was later found to have progressive cirrhosis and portal vein thrombosis in the main portal trunk during the follow-up period. Although pre-transplantation imaging studies showed only a small recurrent HCC with no PVTT in the cirrhotic liver, pathological examination did not reveal any viable HCC or PVTT after LDLT.

### 3.2. Outcome Analysis

**Figure 1:**
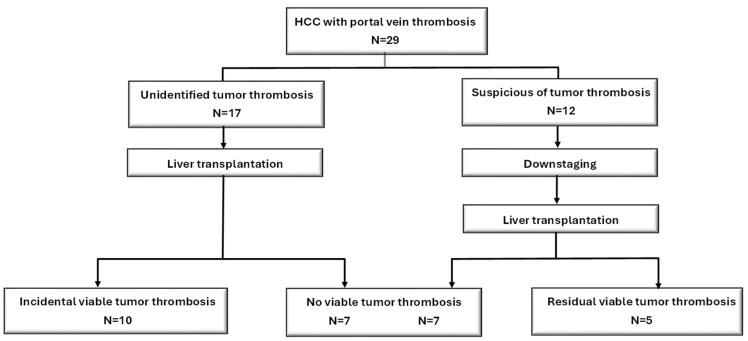
Flow diagram of patients with hepatocellular carcinoma (HCC) and portal vein thrombosis who underwent liver transplantation.

The clinicopathological characteristics of the patients, characterized by a pre-transplant PVTT diagnosis, are summarized and compared in Table 2. The two groups had comparable clinical features. However, 12 patients suspected of having PVTT received radiation and/or systemic therapies for downstaging before LT. Eight patients (27.6%) received DDLT, and twenty-one patients (72.4%) were LDLT. There was no significant difference in terms of waiting period between the two groups (*p* = 0.313). However, the waiting period of DDLT (median: 29.3 months) was significantly longer than LDLT (median: 3.1 months) in the 29 patients (*p* = 0.009). Pathological examination of the explanted liver showed viable HCC in the portal vein thrombus in five patients. Among the patients who were PVTT-free before LT, 10 had viable HCC in the portal vein thrombus (Figure 1).

The median follow-up period for the entire cohort was 66.7 months (range: 0.6–148.8 months) after LT. During this period, seven (24.1%) patients experienced HCC recurrence. The median time to HCC recurrence was 18.0 months (range: 6.9–87.9 months) post-transplant. The clinical characteristics of the seven patients with post-transplant HCC recurrence are summarized in Table 3. All the HCC recurrences were extrahepatic metastases. Overall, twelve patients died at the end of this study, including six who died from HCC recurrence. The remaining six patients died without recurrence. One patient who experienced recurrence 18.0 months after LT is alive.

### 3.3. Survival of Patients

**Figure 2:**
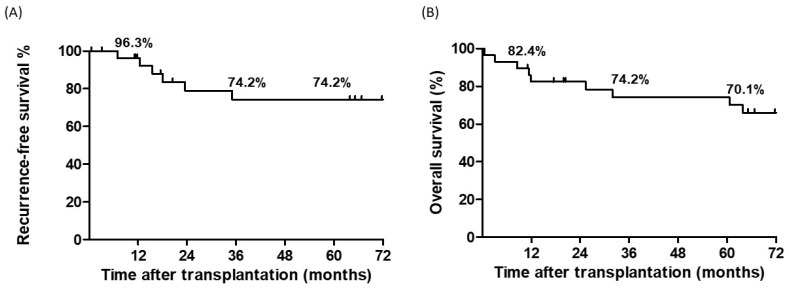
Kaplan–Meier survival curves in patients (*n* = 29) with advanced hepatocellular carcinoma (HCC) and portal vein thrombosis following liver transplantation. (**A**) Recurrence-free survival curve. (**B**) Overall survival curve.

**Figure 3:**
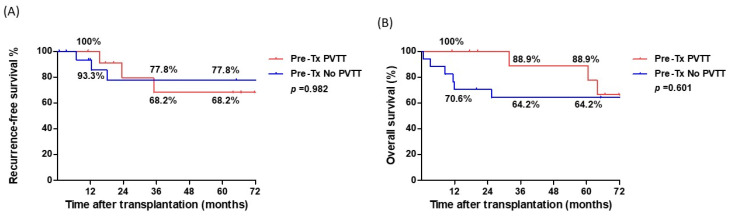
Comparison of cumulative survival curves based on pre-transplantation detection of portal vein tumor thrombosis (Pre-Tx PVTT). (**A**). Recurrence-free survival curves (*p* = 0.982). (**B**). Overall survival curves (*p* = 0.601). Pre-Tx PVTT, *n* = 12; Pre-Tx No PVTT, *n* = 17.

Among the 29 patients with HCC and PVT, the 1-, 3-, and 5-year RFS rates after LT were 96.3%, 74.2%, and 74.2%, respectively, whereas the 1-, 3-, and 5-year OS rates were 82.4%, 74.2%, and 70.1%, respectively (Figure 2). Patients with pre-transplant PVTT who underwent downstaging therapy were further compared with those who were PVTT-free pre-transplant. RFS and OS rates were not significantly different between the two groups (Figure 3). The 5-year RFS and OS rates were 68.2% and 77.8%, and 88.9% and 64.2%, respectively.

**Figure 4:**
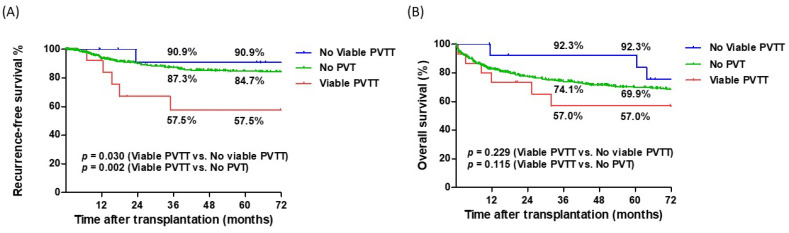
Comparison of cumulative survival curves according to HCC patients with or without portal vein thrombosis (PVT). (**A**). Patients with HCC plus viable portal vein tumor thrombosis (PVTT) have a significantly inferior recurrence-free survival curve compared with the other two groups (*p* = 0.006). (**B**). Overall survival curves have no significant difference in the three groups (*p* = 0.251). No Viable PVTT, *n* = 14; Viable PVTT, *n* = 15; No PVT, *n* = 472.

Patients with macrovascular invasion, indicated by viable HCC in the portal vein, showed a significantly lower RFS than those without viable PVTT (*p* = 0.030) and other HCC patients (*n* = 472) without PVT (*p* = 0.002) (Figure 4A, *p* = 0.006). However, the comparison of OS was a not significant difference (Figure 4B, *p* = 0.251). Among patients without viable PVTT, the 1-, 3-, and 5-year RFS rates post-LT were 100%, 90.9%, and 90.9%, respectively, whereas the OS rates were 92.3%, 92.3%, and 92.3%, respectively. In contrast, for patients with viable PVTT, the 1-, 3-, and 5-year RFS rates after LT were 92.3%, 57.5%, and 57.5%, respectively, whereas the OS rates were 73.3%, 57.0%, and 57.0%, respectively. Among other HCC patients without PVT, the 1-, 3-, and 5-year RFS rates and OS rates after LT were 93.8%, 87.3%, and 84.7% and 83.0%, 74.1%, and 69.9%, respectively.

**Figure 5:**
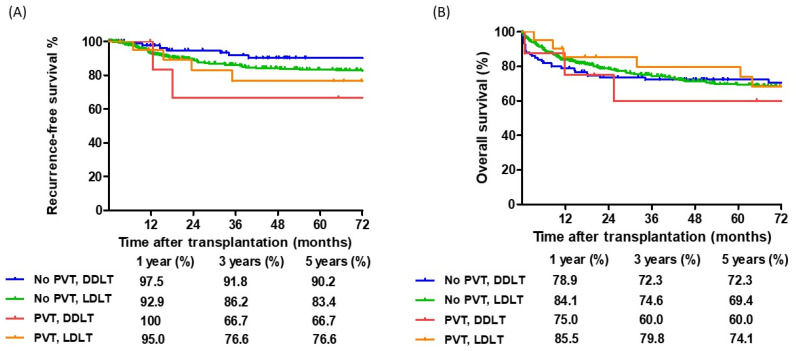
Comparison of cumulative survival curves for deceased donor liver transplantation (DDLT) and living donor liver transplantation (LDLT) across the entire cohort. (**A**) Recurrence-free survival curves (*p* = 0.219). (**B**). Overall survival curves (*p* = 0.883). No PVT DDLT, *n* = 100; No PVT LDLT, *n* = 372; PVT DDLT, *n* = 8; PVT LDLT, *n* = 21.

Moreover, the study included both DDLT and LDLT, which showed a significant difference in waiting periods. Consequently, the outcomes in terms of DDLT and LDLT were further analyzed. However, no significant differences were identified when comparing the RFS (*p* = 0.219) and OS (*p* = 0.883) rates in this study (Figure 5).

## 4. Discussion

LT has become a standard treatment for many end-stage liver diseases, as well as for certain groups of patients with HCC. Despite their strict requirements, the Milan and UCSF criteria remain the gold standard for assessing HCC suitability for LT. However, advancements in medicine have enabled the transplantation community to overcome many challenges related to LT. As a result, expanding the criteria for LT for HCC has been a longstanding and critical issue. Notably, expanded criteria regarding the size and number of HCC tumors have shown favorable outcomes in many major transplant centers worldwide [18,19,20,21,22]. However, extending the criteria beyond vascular invasion, such as in cases of PVTT, remains controversial and has only been reported by a few transplantation centers [23,24,25]. The study retrospectively analyzed the outcomes of LT in patients with HCC and portal vein thrombosis. These results indicate that residual viable tumor in the thrombus may lead to poor outcomes after LT and should be approached with caution.

Generally, PVTT may be encountered in LT in patients with HCC in three major scenarios. First, a tumor thrombus was incidentally discovered in the explanted liver during the histological examination. Second, patients with preexisting PVTT underwent downstaging treatment to meet the LT criteria. Third, patients with preexisting PVTT underwent LT without downstaging treatment. In fact, HCC associated with PVTT should be excluded from LT based on the current traditional and expanded criteria. However, HCC is often associated with cirrhosis, which can lead to portal hypertension as well as the formation of portal vein thrombosis [26]. Therefore, distinguishing between benign portal vein thrombosis and PVTT whenever thrombosis develops in the portal vein can be challenging in clinical practice. Currently, cross-sectional imaging is the primary tool for assessing the nature of portal thrombi. Although certain criteria can help characterize PVTT in HCC, their predictive accuracy remains suboptimal [27]. Therefore, tumor thrombosis may still be encountered in LT for HCC, as demonstrated in this study. Moreover, expanding the transplant criteria too broadly may increase the risk of undetected PVTT pre-transplant.

However, HCC with PVTT can still be considered for LT if the appropriate therapy can achieve successful downstaging. Currently, standard protocols for downstaging HCC with PVTT prior to LT are lacking. Generally, a range of available therapies, alone or in combination, can be used to downstage HCC with PVTT [28,29,30,31]. Emerging evidence suggests that transarterial radioembolization (TARE) and external radiation therapy may play a crucial role in treating patients with HCC and PVTT [8,32,33]. TARE with yttrium-90 provides both internal radiotherapy and a microembolic effect. Additionally, external radiation therapy, which delivers higher radiation doses to target regions without causing damage to adjacent liver tissues, could be a valuable treatment for PVTT. Several studies have demonstrated that additional radiotherapy targeting PVTT can improve the prognosis of patients with HCC [10,11,34,35]. Specifically, combining additional radiotherapy and multimodality treatment offers a promising strategy for downstaging HCC with PVTT before LT [9,36]. In this study, stereotactic body radiation and proton beam therapies were used to downstage PVTT. Although pre-transplantation imaging examinations indicated successful downstaging, viable tumor cells remained in the portal vein after microscopic histological examination of the explanted liver.

Moreover, current treatment guidelines recommend systemic therapy for managing HCC with PVTT [6]. Particularly, systemic therapy for advanced HCC has achieved notable progress in the last decade [37,38,39,40]. Accumulating evidence has shown the promising therapeutic effects of ICIs in patients with advanced HCC. However, the effectiveness of these novel systemic therapies for patients with HCC and PVTT in real-world clinical practice remains limited, particularly regarding downstaging patients to meet the LT criteria. In addition, the risk of graft rejection should be considered when using ICIs in patients with HCC undergoing LT [41,42,43]. In this study, two patients who received short-term ICI treatment before transplantation did not experience lethal graft rejection after LT. These few cases are not sufficient to confirm the safety of ICIs before LT. However, with the rapid increase in the use of ICIs to downstage HCC for LT, most experiences indicate that the time interval between ICIs and LT may play a crucial role in the occurrence of lethal graft rejection after LT [44].

In this study, pathological examination revealed viable PVTTs in half of the patients. Theoretically, the presence of viable PVTT suggests circulating cancer cells may contribute to poor post-LT outcomes. Indeed, tumor recurrence with systemic spread was significantly associated with PVTT viability in this study. Although OS did not significantly differ between the two groups regarding viable PVTT, tumor recurrence remained a significant cause of death and poor prognosis in patients with HCC undergoing LT. However, accurately detecting viable PVTT and staging HCC through preoperative imaging as the exclusion criterion is perhaps challenging. The presence of an underlying cirrhotic liver, along with additional locoregional therapies, complicates the interpretation of radiological images. As shown in our series, LT was recommended for patients only if their HCC met the UCSF criteria based on imaging examinations. However, 28.3% of patients exceeded the UCSF criteria based on pathological results, indicating that not all pre-transplant radiological images align with pathological staging. Instead, other factors such as tumor variation and tumor marker behavior during bridging or downstaging therapy should be considered. If PVTT is excluded from LT, an integrated approach for accurately diagnosing tumor thrombi may be necessary. Additionally, no effective adjuvant therapy currently exists to prevent HCC recurrence after LT. Therefore, performing LT in patients with HCC and PVTT requires careful, individualized, and case-by-case assessment to identify suitable candidates.

## 5. Conclusions

This study was limited by its retrospective design and small sample size. Although generalizations from these limited experiences could not be easily achieved, several significant findings may inform the management of patients with HCC and PVTT using LT. Therefore, LT could still be considered for patients with HCC and PVTT after appropriate downstaging treatment. However, caution is warranted, as viable PVTT may lead to unsatisfactory post-LT outcomes. Additionally, many unknowns related to LT for HCC with PVTT need to be explored in the future. Based on the current evidence, LT for HCC with PVTT remains controversial.

## Figures and Tables

**Table 1:** Clinical features of patients with HCC who underwent liver transplantation.

Characteristics	HCC with PVT		*p* Value
	Yes (*n* = 29)	No (*n* = 472)	
Age (years), median (range)	55.1 (42.1–68.6)	57.0 (13.0–70.0)	0.544
Sex, Male:Female	24:5	373:99	0.814
Viral hepatitis			0.664
HBV	18	299	
HCV	6	113	
HBV + HCV	1	24	
Alcohol	3	35	0.474
AFP (ng/mL), median (range)	9.0 (1.3–1384)	12.0 (1.7–18,250)	0.665
MELD score, median (range)	12 (6.0–25)	13 (5.0–17.0)	0.270
Child–Pugh Class			0.714
A	13	205	
B	11	156	
C	5	111	
Type of transplantation			0.415
DDLT	8	100	
LDLT	21	372	
Tumor Number, median (range)	2 (0–8)	2 (0–22)	0.476
Maximum tumor (cm), median (range)	3.3 (0–11.1)	2.5 (0–11.2)	0.031
Pathologic UCSF criteria			0.007
Within	10	349	
Beyond	19	123	

HCC, hepatocellular carcinoma; PVT, portal vein thrombosis; HBV, hepatitis B virus; HCV, hepatitis C virus; AFP, alpha-fetoprotein; MELD, model for end-stage liver disease; DDLT, deceased donor liver transplantation; LDLT, living donor liver transplantation; UCSF, University California of San Francisco.

**Table 2:** Clinical features of HCC associated with portal vein thrombosis.

Characteristics	Pre-Transplantation Diagnosed PVTT		*p* Value
	Yes (*n* = 12)	No (*n* = 17)	
Age (years), median (range)	53.6 (42.1–67.2)	55.5 (43.6–68.6)	0.492
Sex, Male:Female	10:2	14:3	1.000
Viral hepatitis			0.573
HBV	8	10	
HCV	2	4	
HBV + HCV	1	0	
Alcohol	0	3	0.124
Platelet (1000/μL), median (range)	99 (54.0–255.0)	70 (14.2–223.0)	0.570
Liver enzyme, median (range)			
AST (U/L)	54 (23–235)	56 (23–332)	0.582
ALT (U/L)	46 (20–165)	31 (12–201)	0.343
Alk-p (U/L)	126 (52–577)	123 (60–205)	0.228
Total Bilirubin (mg/dL)	1.2 (0.3–15.1)	2.1 (0.3–15.8)	0.742
Albumin (g/dL)	3.6 (2.3–4.6)	3.2 (2.0–4.6)	0.260
ALBI grade			0.534
1	4	3	
2	5	7	
3	3	7	
Child–Pugh class			0.099
A	8	5	
B	2	9	
C	2	3	
MELD score, median (range)	7.5 (6.0–23.0)	13.0 (7.0–25.0)	0.259
AFP (ng/mL), median (range)	10.1 (1.3–562)	4.9 (2.0–1384)	0.932
Extent of portal vein thrombosis			0.790
Second order branch	4	7	
First order branch	5	5	
Main portal trunk	3	5	
Pre-LT TACE			1.000
<4	8	11	
≥4	4	6	
Pre-LT RFA	2	5	0.664
Systemic therapy			0.037
TKIs	2	0	
ICIs	2	0	
Radiation therapy			<0.001
Radiotherapy	9	0	
Proton therapy	3	0	
Type of transplantation			0.926
DDLT	1	7	
LDLT	11	10	
Tumor number, median (range)	1 (1–7)	2 (0–8)	0.563
Maximum tumor (cm), median (range)	3.4 (0.6–11.1)	2.9 (0–8.3)	0.369
Total tumor size (cm), median (range)	5.4 (0.6–15.0)	4.4 (0–19.2)	0.659
Pathologic UCSF criteria			0.912
Within	4	6	
Beyond	8	11	

HCC: hepatocellular carcinoma; PVTT: portal vein tumor thrombosis; HBV: hepatitis B virus; HCV: hepatitis C virus; AST, aspartate aminotransferase; ALT, alanine aminotransferase; Alk-p, alkaline phosphatase; ALBI, Albumin–Bilirubin; MELD, model for end-stage liver disease; AFP: alpha-fetoprotein; LT: liver transplantation; TACE: transarterial chemoembolization; RFA: radiofrequency ablation; TKIs: tyrosine kinase inhibitors; ICIs: immune checkpoint inhibitors; DDLT: deceased donor liver transplantation; LDLT: living donor liver transplantation; UCSF, University California of San Francisco.

**Table 3:** Clinical features of patients with HCC recurrence after liver transplantation.

Patient No.	Age/Sex	Extent of PVT (Branch)	Viable PVTT	Time to Recurrence (Months)/Location	Follow-Up (Months)/Outcomes
1	51/M	First	No	23.5/Bone, peritoneum	60.6/Dead
2	48/M	Second	Yes	15.4/Lung	31.9/Dead
3	63/M	Second	Yes	12.5/Lung	25.4/Dead
4	69/M	First	Yes	6.9/Peritoneum	8.5/Dead
5	53/M	Second	Yes	87.9/Cardiophrenic LNs	95.3/Dead
6	52/F	Second	Yes	35.0/Mediastinal and para-aortic LNs	77.8/Dead
7	56/F	First	Yes	18.0/Bone	20.0/Alive

HCC: hepatocellular carcinoma; PVT: portal vein thrombosis; PVTT: portal vein tumor thrombosis; LNs: Lymph nodes; M: male; F: female.

## Data Availability

All data were included in this study.
